# A cautionary note on testing latent variable models

**DOI:** 10.3389/fpsyg.2015.01715

**Published:** 2015-11-06

**Authors:** Ivan Ropovik

**Affiliations:** Department of Preschool and Elementary Education and Psychology, Faculty of Education, University of PresovPresov, Slovakia

**Keywords:** structural equation modeling, confirmatory factor analysis, model fit, chi square test, approximate fit indices

## Abstract

The article tackles the practice of testing latent variable models. The analysis covered recently published studies from 11 psychology journals varying in orientation and impact. Seventy-five studies that matched the criterion of applying some of the latent modeling techniques were reviewed. Results indicate the presence of a general tendency to ignore the model test (χ^2^) followed by the acceptance of approximate fit hypothesis without detailed model examination yielding relevant empirical evidence. Due to reduced sensitivity of such a procedure to confront theory with data, there is an almost invariable tendency to accept the theoretical model. This absence of model test consequences, manifested in frequently unsubstantiated neglect of evidence speaking against the model, thus implies the perilous question of whether such empirical testing of latent structures (the way it is widely applied) makes sense at all.

## Introduction

One of the fundamental issues of empirical research in psychology is the measurement of variables defined within a given research problem. If the researcher discards the doctrine of operationalism, which equates the theoretical construct with its measure, the most common alternative measurement theory to be applied is the *latent variable theory*. Here, the theoretical construct is defined as a latent variable which acts as a directly unobservable causal determinant of the variance in a set of manifested variables (Borsboom et al., 2003). Within this paradigm, the researcher postulates a theory in the form of a statistical model and tests whether the theory models the observed reality (the data) well. Only if the observation and the theory (data and model) match each other, is it justified to regard the observation as the measurement of theoretical constructs (Borsboom, 2005) and proceed to the interpretation of relationships between those constructs. The primary statistical tool within this measurement theory is the *structural equation modeling* (SEM) and its special case, *confirmatory factor analysis* (CFA). As early as in 2001, SEM was the most widely used multivariate technique in psychology (Hershberger, 2003) and in recent years, it has become a standard in solving multivariate research problems in most of the social sciences.

To put it simply, this (dis)confirmatory technique allows testing of several postulated hypotheses simultaneously. Employing iterative estimation, it tries to find a unique set of implied parameters (variances, covariances) that would match those in the matrix of observed covariances as much as possible. In such a manner, it can be tested whether the model-implied relations between latent and manifested variables correspond with the existing relations observed in the data. Within SEM, the only statistical test of model-data fit is the *chi-square test* (χ^2^, actually, a family of tests). It tests the null hypothesis that the model-implied covariance matrix **Σ**(**𝜃**) does not significantly differ from the matrix of observed covariances **S**, i.e., that the residuals are not statistically different than zero. A high value of χ^2^ (relative to the model’s *df*) associated with *p* > 0.05 means the following: Given that the null hypothesis of no model-data difference is true, the observed discrepancies between the model and the data are too big to be caused by random fluctuations due to sampling error alone, indicating the presence of a systematic misspecification in the tested model. Logically, following such an indication, the researcher should try to find the misspecification errors that are present in the model in order to achieve convergence between scientific explanation and the principle of phenomena under study.

However, it is a widespread practice in social sciences to ignore a significant χ^2^ and point to the theoretical absurdity of the exact-fit hypothesis postulated by the χ^2^ test (allowing only for non-zero deviations due to sampling error) or to some of the technical properties of the χ^2^ test. These include high power with rising *N*, rising reliability of indicators and higher communalities (Miles and Shevlin, 2007; Heene et al., 2011), sensitivity to distributional properties of observed variables (Fouladi, 2000) or inflated Type I error rate with rising complexity of the model as a function of the size of the covariance matrix (Moshagen, 2012).

What usually comes next is the judgment whether the model approximates the data that is based almost exclusively on so-called *approximate fit indices* (AFI). If the chosen indices exceed some conventional thresholds (see Hu and Bentler, 1999), the model is accepted as adequate (or approximately true) and, based on model parameters, conclusions regarding the nature of studied phenomena are drawn. The practice outlined above can, however, lead to questionable conclusions.

This paper extends on a series of papers published in a special issue of the *Personality and Individual Differences* (Vernon and Eysenck, 2007) on model testing in SEM followed by an extensive discussion of this topic on SEMNET (a web discussion group devoted to SEM). The aim of the paper is to reflect on the progress of the issues connected with testing of latent models and to analyze recent studies in the field of psychology that model latent theoretical structures with respect to the applied testing practices.

To date, several studies documented practices regarding the use of SEM in various fields of social sciences and found several issues of concern. Namely, (1) little attention paid to distributional assumptions, (2) lacking theoretical justification and history of *post hoc* model modifications, most often involving dropping indicators, allowing cross-loadings and correlated error terms, (3) confirmation bias, (4) the use of inadequate estimation method, (5) the failure to recognize the existence of equivalent models, (6) poor justification of causal inferences, and (7) selective reporting and the overall lack of clarity in reporting (Breckler, 1990; DiStefano and Hess, 2005; Guo et al., 2009; Jackson et al., 2009; Nunkoo et al., 2013). In comparison to these past studies, the current study focuses primarily on testing and the assessment of model fit. The emphasis laid on latent model testing (even at the expense of other steps of SEM like specification, identification, modification or interpretation of the model) stems from the fact that it is only empirical *testing* (unlike in exploratory latent techniques like EFA) that is able to objectively and formally compromise the postulated latent model as well as the implications and predictions based on that model. The researcher’s attitude towards testing may be distorted by fallacious interpretations of some conceptual and technical principles; this may then open the gate to subsequent acceptance of possibly flawed psychological models and measures. Based on the sometimes seemingly unquestionable rigor of confirmatory techniques like SEM or CFA, these models and measures gain a lot of credibility, some of which may be unjustified. The treatment throughout is deliberately kept nontechnical to get the message to those who probably wouldn’t read it otherwise.

## Materials and Methods

In order to assemble a representative sample of empirical studies employing some confirmatory technique of modeling latent structures, 11 journals from the field of psychology were chosen. These included the *British Journal of Psychology* (two studies), *Journal of Research in Personality* (11), *Journal of Occupational and Organizational Psychology* (10), *Journal of Research on Adolescence* (6), *Developmental Science* (3), *Intelligence* (12), *Early Childhood Research Quarterly* (7), *European Journal of Personality* (10), *Studia Psychologica* (3), *Journal of Environmental Psychology* (5), and *Journal of Experimental Child Psychology* (6). These journals represent a wide range of psychology subfields (including also journals of general orientation) and associated impact factors (from journals with lower impact to top-ranked journals), retrieved from SCImago Journal Rank (SCImago, 2007). The inclusion criteria were as follows: (1) The study was published from 2011 to 2013, reflecting recent practices in latent variable modeling; (2) the study employed some kind of latent variable modeling technique like SEM, CFA, or latent growth analysis (the latter two being special cases of SEM). Apart from using these target words for a *full text* search (i.e., SEM did not have to be the primary focus of the study), a search (as a Boolean phrase) for at least one of the following technical terms contained in these kinds of analyses was also conducted: RMSEA or CFI or TLI or GFI or NFI or SRMR or AIC or BIC (the acronyms of Root Mean Square Error of Approximation, Comparative Fit Index, Tucker-Lewis Index, Goodness-of-Fit Index, Normed fit Index, Standardized Root Mean Square Residual, Akaike Information Criterion, Bayesian Information Criterion). The search resulted in a universe of 424 studies. To ensure that the sample represents the universe of selected studies well, a percentage proportion for every journal was computed (number of studies for every journal is given in parentheses above). The proportional composition of the sample (*N* = 75) reflected the universe from which the studies for analysis were drawn. Within each journal, a random number generator was used to choose the studies for the sample. In the next phase, all the selected studies were screened to see whether they really match the criterion of applying some kind of confirmatory latent variable modeling technique. This resulted in the exclusion of 10 studies (one study was a review, nine studies employed non-latent path analysis). These studies were replaced using random sampling from the same journal. If the study included several independent substudies, the study to be analyzed was chosen by a random draw. Provided that the study reported the testing of several models, the choice of the model for review followed two criteria: (1) most of the interpretations were based on that model and its parameter estimates (expected to be the best fitting one). If not clearly decisive, (2) the model with better fit (by χ^2^) was chosen.

The analysis focused on the following aspects: (1) Aspects determining the fulfillment of distributional assumptions for using SEM (i.e., the sample size, the assessment of univariate and multivariate normality), (2) aspects connected with model testing (the employed fit function, the use of χ^2^ test and AFI, the assessment of local fit by inspecting residual matrix, testing of alternative models), and (3) outcome of model testing and the interpretation of the model (interpreted adequacy of the model, the reasons for model acceptance, the presence of *post hoc* modifications, the reasons for the eventual disregard of the model test, reporting of data and results).

For further analyses, the full dataset can be found in Supplementary Material.

## Results

Out of 75 selected studies, 44% used CFA, 45% used SEM (models which, apart from the CFA measurement models, involve regression paths between latent variables), and 11% employed latent growth analysis. In 66% of the studies, only questionnaire or rating scales data were analyzed, 28% employed only tests, 5% made use of both questionnaires and tests, and there was also one (1%) meta-analysis analyzing secondary data. The central tendency in sample sizes of the selected studies was at *Mdn* = 308 (*range* = 8658), including six studies using secondary data. The researchers most frequently reported the use of the following software packages: M*plus* (32%), Amos (24%), and Lisrel (16%). In justification of the normal theory-based estimation method, the assumption of univariate normality of variables was examined in 24% of the studies. The assumption of multivariate normality was explicitly examined or taken into account (by using robust estimation methods – Robust maximum likelihood (RML), Satorra–Bentler χ^2^) in 27% of the selected studies.

Regarding the testing of the model fit, as can be seen in **Table 1**, the most frequently used fit function was the method of maximum likelihood (ML). What is notable is the fact that 43% of the studies did not report which fit function was used to fit the model to the data. Model χ^2^ with degrees of freedom (*df*) and associated significance level was reported in 41% of the studies. For all of the studies (100%) lacking a report of *p-*value for the χ^2^ of the postulated models but reporting the *df* (40%), the χ^2^ would have been significant. Most of the studies (91%) reported more than two AFI. The most frequently reported ones were RMSEA, CFI and SRMR (91, 89, and 51%, respectively). Regarding the cut-off criteria for the AFI, 17% of the studies reported to follow the conventional criteria recommended by Hu and Bentler (1999). The author’s custom selection of less strict criteria could be seen in 30% of the studies and 53% of the studies did not explicitly report the exact criteria applied. In the case of significant χ^2^, the decision on the adequacy of the tested models was usually based on AFI, since the examination of local fit by inspecting the residual matrix was reported only in 3% of the studies. Residuals were not reported in any of the models with a non-significant χ^2^.

**Table 1:** Aspects of testing latent models.

Study characteristics		%
Fit function	Maximum likelihood (ML)	40
	Robust maximum likelihood (RML)	13
	Weighted least squares (DWLS)	4
	Not reported	43
χ^2^ test^∗^	Reported values of χ^2^, *df*, and significance	41
	χ^2^ not reported	11
	*df* not reported	21
	*p* not reported	40
	Satorra–Bentler χ^2^	12
The usage of approximate fit indices (AFI)^∗^	RMSEA RMSEA confidence intervals	91 24
	χ^2^/*df*	20
	CFI	89
	TLI (NNFI)	37
	GFI	13
	NFI	5
	SRMR	51
	AIC	13
	BIC	8
	Other (PCFI, PGFI, IFI, ECVI, BCC)	<3
Alternative models	Yes	64
	Not reported	36
*Post hoc* modifications	Yes	46
	No	41
	Not clear	13


*Overlapping categories are marked with an asterisk (^∗^).*

Among the most important aspects of every analysis are the consequences of model testing and the interpretation of the model (does the model fit the data so that it is possible to interpret model parameters?). Here, almost all of the studies (97%) reported at least one model that was considered adequate and served as the basis for further interpretations. However, out of these models (*N* = 73), 80% did not fit according to the model test and the decision to retain and interpret the model was probably based on some other criteria, particularly AFI (40% of these studies just provided the fit indices and noted that the model fits but did not explicate the basis for such conclusion). Only 3% of the studies (*N* = 2) concluded that the best model does not fit by any measures, however, one of them proceeded to the interpretation of model parameters anyway. Overall, 80% of the studies ignored the χ^2^ model test either by ignoring the associated significance of *p* > 0.05, or by not reporting it at all. Out of these studies (*N* = 60), 75% did not mention the reasons for ignoring the model test. On the other hand, the explicitly stated reasons given by the authors for ignoring the outcome of the model test can be summarized as follows: (1) χ^2^ is overly sensitive to sample size, (2) χ^2^ penalizes models when the number of variables gets high, (3) the exact fit hypothesis is nonsensical and (4) there is a broad consensus on the preference of the use of AFI.

Regarding the issues of reporting SEM analyses, 57% of the studies provided the data needed for the sake of proper replication of conducted analyses (mostly correlation matrix and *SD*’s).

## Discussion

### Disregard for the χ^2^ Test

The most disputable tendency observed in analyzed studies is the disregard for the result of model test. Usually, the result that was ignored was shown to be invariably the one that spoke against the theory being tested. If a model significantly departs from the observed data, it seems that the researchers are much more likely to slip into a kind of argumentum ad hominem fallacy and blame the integrity of the test procedure than what should be questioned in the first instance – the model. However, the only message that a significant χ^2^ tells is just this: “Take a good look at that model, something may be wrong here.”

If stated explicitly, the most frequent justification of disregard for the χ^2^ model test was its undeniable sensitivity to the sample size. In general, χ^2^ actually gets computed as (*N–*1)*F*_min_, where *N* is the sample size and *F*_min_ is the minimum of the fit function which minimizes the discrepancies between the matrices **Σ**(**𝜃**) and **S**. If the model does not correspond with the data in some way, the sample size multiplies the detected discrepancies between the model and the data. It is true that with large samples, even trivial discrepancies caused by theoretically irrelevant causal factors can produce a significant misfit. However, a conclusion that a significant χ^2^ based on a large sample is a product of its excessive sensitivity is actually another sort of formal logical fallacy, namely affirming the consequent. With large enough sample, the statement “if the misspecification is trivial, χ^2^ is significant” is true. However, if the misfit is significant, it does not imply that the misspecification is trivial. The model test failure can be caused by one or even several serious misspecifications, regardless of sample size (McIntosh, 2012; Hayduk, 2014a).

Instead of perceiving statistical power (primarily but not exclusively driven by sample size) as the ability of the test to reveal the discrepancies between the model and the data, it is frequently regarded as the main reason for disregarding the model test. Now here comes the question: If the outcome of a hypothesis test (especially the *H0* rejection) is ignored, what was the aim of doing it? Although several studies ascribed model failure to the high statistical power of the χ^2^ test, not even one study empirically verified this claim by power analysis (see Saris and Satorra, 1993; MacCallum et al., 1996; Muthén and Muthén, 2002). One could thus avoid an *a posteriori* refusal of the model test outcome even before any data are collected and, in a way, define what degree of theory-data misfit could be ascribed to sampling error fluctuations and peripheral causal effects.

It can be concluded that even though the χ^2^ test is by far not flawless, with adequate power that allows the model test to miss only sufficiently peripheral causal effects (verified by an a priori power analysis), it is nowadays the best, formally definable protection against ill-specified theories represented by latent models. In addition, its sensitivity to misspecifications easily outperforms all the other fit indices (Marsh et al., 2004; McIntosh, 2012).

### Approximate Fit Indices

On the other hand, the structural equation model is usually a system of many hypotheses, every single one of which can be true or false. The rejection of the model’s exact fit hypothesis, however, does not necessarily falsify all the hypotheses comprising the model. A significance of χ^2^ can have several causes. As well as resulting from the invalidity of any of the postulated hypotheses, it can also be caused by the heterogeneity of the studied sample (i.e., the underlying causal model varies between subgroups of subjects, or there are some intervening within-subject factors), the observations may not be truly independent, or in some cases it can be a severe violation of multivariate normality assumption (Fouladi, 2000; Yuan et al., 2005). Any one of these eventualities coupled with adequate power can lead to the rejection of the null hypothesis of exact fit. A strict requirement for a non-significant χ^2^ may appear absurd, and maybe one has to accept the fact that all models in social sciences only approximate the reality and so are bound to be wrong to a certain degree (Browne and Cudeck, 1992; MacCallum et al., 1996; Mulaik, 2009). In this context, do the AFI allow for the renunciation of such a philosophically and technically questionable χ^2^ test?

Unfortunately, the impression that the AFI (especially the way they are used) are loaded with lesser problems is just illusory. Despite the fact that the term AFI represents a conceptually heterogeneous group of fit indices, none of them offer critical values and their associated significance. Their distributions (except for RMSEA) are unknown (Yuan, 2005), and although most of them (the group of absolute fit indices) are, apart from sample size and *df*, a function of the χ^2^, these indices have no established statistical basis and do not represent a formal test of the model (Hayduk et al., 2007).

In theory, all the AFI can be used for hypotheses testing, but what is needed are the cut-off values indicating acceptable approximate fit. Within the sample of analyzed studies, the authors applied a wide range of criteria, some of them more, some less strict. These criteria (e.g., Hu and Bentler, 1999) are mostly based on Monte-Carlo simulations and reflect a limited array of situations. Despite the warnings of their authors, AFI criteria are frequently regarded as golden axiomatic rules having universal validity. Follow-up simulation studies, however, clearly showed that: (1) It is not possible to establish universal cut-off criteria regardless of the character of the model tested (Marsh et al., 2004; Beauducel and Wittmann, 2005; Fan and Sivo, 2005; Sharma et al., 2005; Yuan, 2005); (2) with several frequently used AFI, the probability of correctly rejecting misspecified models systematically decreases with growing *N* (Marsh et al., 2004; Sharma et al., 2005) which is, paradoxically, the very opposite as with the χ^2^ test, and (3) the behavior of the AFI is highly unpredictable in the presence of severe misspecifications (Fan et al., 1999), which also holds under various degrees of correlated errors (Heene et al., 2012).

Regarding the AFI, an exemplary case of their problematic usage is the χ^2^/*df*. One fifth of the analyzed studies based their decision to accept the model also on χ^2^/*df* although the author of this index withdrew his recommendation for its usage quite a long time ago (see Wheaton, 1987). Apart from that, several simulation studies clearly demonstrated that the χ^2^/*df* indicated a good fit of the model and the data despite severe model misspecifications (Kaplan, 1988; Steiger, 2000). Saris et al. (1985) found that if the model did not fit the data, the authors regarded almost every sample size as too large for the χ^2^ and almost every χ^2^/*df* ratio as acceptable. Here, among the analyzed studies, none discarded the model due to a high χ^2^/*df* ratio and there were some that did not even see ratios as large as e.g., 5, 10, 19, or even 20 as problematic, although there is a massive degree of non-centrality. Rather loose and confirmationist practices can also be seen with other AFI where one can often find the reference for a convenient cut-off value (see Hooper et al., 2008).

As well as the above mentioned issues with the AFI, there is another that is also important. Some studies (e.g., Hayduk and Glaser, 2000; Hayduk et al., 2005; Saris et al., 2009) suggest that acceptable fit indices may (but do not have to) reflect an inconsequential misspecification since a pattern of even rather moderate residuals can be a sign of serious misspecification. Therefore, satisfactory values of AFI indicating close fit (e.g., CFI ≥ 0.95) do not ensure that the model failed the χ^2^ test because of a trivial misspecification (Olsson et al., 2000; Tomarken and Waller, 2003; McIntosh, 2012; Hayduk, 2014a).

### The Consequences of Disregarding the Model Test

Disregarding the outcome of the χ^2^ test and, based on the AFI, assuming the triviality of the misspecification without a detailed inspection of the model, can in some cases lead to an unjustified acceptance of an incorrect theory. There is but another consequence that is not a possibility, but rather a certainty.

Namely, one of the assumptions of iterative estimation procedures used in SEM is that the model is fully correctly specified. If that assumption is violated, even one misspecification can spread throughout the model and affect all the estimated parameters (Kaplan, 1988). If such a model contains several misspecifications, their effects can be combined in unpredictable directions. It follows that if the researcher does not trust the significant χ^2^, he/she should not trust the model parameter estimates either (Bollen et al., 2007; Antonakis et al., 2010). Actually, the χ^2^ employs the same statistical principles on which the parameter estimation is based (Bollen et al., 2007). If the χ^2^ is significant, it means that the fit function was unable to find a set of parameters that would fit the data sufficiently well. A significant χ^2^ (especially with high degree of non-centrality) thus warrants that the parameter estimates are inaccurate at best and, at worst, squarely wrong.

To be able to interpret the estimated parameters and carry out further procedures (e.g., the testing of measurement invariance, the use of item parcels), the model must be correctly specified in the first place and the χ^2^ is the most direct formal proxy for such an assessment. Although the detection of misspecification is not the only aspect of model assessment (others are, e.g., the examination of predictive power, explanatory parsimony, theoretical backing of direct, and indirect effects), it is the most important one since just one misspecification can possibly affect the parameter estimates throughout the entire model and all the other mentioned aspects of model assessment are based on these estimates (Hayduk et al., 2007). None of the analyzed studies that interpreted the model parameters despite a significant misfit warned the reader that these interpretations are questionable, because they are based on potentially distorted parameter estimates.

Just to add, some of other problematic practices that could be observed in the reviewed papers include: (1) A badly fitting model is suggested to fit well because it fits better (by Δχ^2^) than a much worse fitting alternative model; (2) depriving the model of its *df* (i.e., testability) to mask misfit by creating item parcels when independent clusters model does not fit the data, or by (3) dropping indicators and correlating error terms until the model fits.

### Suggested Solutions

We have to admit that in social sciences, the model-data exact fit hypothesis postulated by the χ^2^ is actually unrealistic for many typical applications. At the same time, such a conclusion should not be used as a universal argument for the acceptance of any seemingly adequate model without it first being carefully inspected. If the theory fails the empirical testing against the data, the explanation of the model failure (and eventual acceptance of the model) has to go beyond an appeal to fit indices thresholds (Millsap, 2007).

Although the AFI might be useful especially in comparing non-nested models, the danger of the actual practice in their use lies in the fact that it enables one to flexibly label a statistically falsified model as approximately true and does not force the researcher into careful inspection of the detected model-data discrepancies. Opposed to it stands the only statistical test of latent models, the χ^2^ test. That test cannot say whether the model is true (the absence of evidence against the model does not mean that it is correct), but if provided with adequate statistical power (large samples, reliable indicators, large communalities), it is superior to all the other fit indices in detecting problems within the tested model (see Marsh et al., 2004). In fact, carrying out an a priori power analysis helps to specify a target amount of misfit that is acceptable. Although the AFI do essentially the same job without any power analysis, it is impossible to determine what amount of model-data misfit they fail-to-detect.

Model testing is the primary tool in the assessment of model adequacy; however, it is in no way the only one. As was suggested, whether the model fits the data or not (by any global measure, be it χ^2^ or AFI), it is essential to carry out a careful inspection in order to identify eventual misspecifications – especially in underpowered designs (see Chen et al., 2001; McIntosh, 2007; Kline, 2011). Although there is no universal recipe, several approaches has been proposed. The most common is the inspection of the standardized residual matrix, examination of the Lagrange multiplier statistics (modification indices) along with the expected parameter change statistics (Saris et al., 2009), or the equation-by-equation misspecification search (Shipley, 2003; Kirby and Bollen, 2009). Generally, one cannot rely on global indices (e.g., SRMR) since every global index is a reduction of dimensionality, reducing several specific model-data discrepancies into one number (Mulaik, 2009). However, even the inspection of the residual matrix can be quite tricky. Because the residuals (and local fit indices) for a misspecified model are biased as well, only the minimum needed number of theoretically justifiable model modifications should be made, and tested each separately. However, the more data-driven modifications are made, the less probable is the reproducibility of such a model (MacCallum et al., 1992) since they may capitalize on chance variation. Interpretations of such *a posteriori* modification and the related inflation of Type I error probability should, therefore, be always taken into account. On the other hand, in some situations, the data are expected to be too noisy and/or the researcher is not able to meet the overly restrictive requirement of zero cross-loadings of the CFA measurement model. In that case, rather than engaging in many data-driven model modifications, resulting in an unreproducible model under the guise of confirmatory analysis, it is better to explicitly acknowledge the uncertainty and employ a procedure that is suited for that purpose, like the Exploratory SEM (Marsh et al., 2014). In this technique, in addition to or instead of CFA measurement model parts, EFA measurement model parts with factor loading matrix rotations can be used, while still preserving the ability to formulate testable predictions and have the access to all the usual SEM parameters, such as residual correlations, regressions of factors on covariates, and regressions among factors. This ensures that, e.g., the misspecification of zero loadings will not lead to distorted factors with overestimated factor correlations and subsequent distorted structural relations (Asparouhov and Muthén, 2009).

Only if there are (1) no signs of severe local misspecification, (2) no other indications of misfit (convergence problems, improbable signs and values of estimated parameters, collinearity, inflated standard errors, negative variances, standardized solution involving values exceeding the interval (1,–1), empirical underidentification due to near zero intercorrelations of the indicators), and (3) there is adequate power, it is justified to conclude that the theoretical model probably approximates the observed reality and that the model test failure is an artifact of high power.

On the other hand, even in the case of exact fit, it is not guaranteed that the model is a true representation of the data. The interpretation of any unrejected model thus rests on an inference that must be based on strong assumption, namely that there is no other alternative explanation (model) for the data at hand. However, especially for cross-sectional data, such inference cannot be formally justified. Actually, the data usually fit several mathematically equivalent models (which is not a real threat), but sometimes even some theoretically justifiable models (Raykov and Penev, 1999; Tomarken and Waller, 2003). “That is, if a model is consistent with reality, then the data should be consistent with the model. But if the data are consistent with a model, this does not imply that the model corresponds to reality” (Bollen, 1989, p. 68). As was already mentioned, achieving fit is quite easy since every model can be made to fit the data well simply by adding free parameters (Kline, 2011). It is often overlooked that the eventual acceptance of model-data fit hypothesis provides only the evidence in favor of fixed parameters (not to be freely estimated). The free parameters are in fact ignored, since every free parameter (e.g., a regression path) expresses an irrefutable hypothesis that there may or may not be a relationship between given variables. The more free parameters (and less *df*) the model has, the fewer dimensions of data space there are along which a model could be rejected (Mulaik, 2001). The information used for the estimation of free parameters cannot be subsequently used for model testing. It follows that a researcher who intends to test a theoretical model should not weaken it by adding free parameters in an *ad hoc* manner (see Mulaik et al., 1989).

To sum up the recommendations: (1) Under all circumstances, it is important to carefully evaluate and properly report the result of the model test (see Kline, 2011; Boomsma et al., 2012, for SEM reporting guidelines); (2) especially in the case of a significant χ^2^ test (the model departs from the observed reality), it should not be concluded that the model approximates the data and ascribe the model test failure to statistical power without a careful inspection of local fit and reporting of achieved power. Satisfactory values of AFI do not guarantee that the misspecification is of trivial magnitude and character; (3) a significant χ^2^ tells that the parameter estimates are inaccurate at best, and possibly wrong. All interpretations based on these estimates should take this fact into account. In any case, one should examine both the causes and consequences of a failed model test.

## Conclusion

The study at hand discussed the practices that are applied in the testing of latent models. But is such a specific treatment of any broader relevance? For a layman reader, it may seem that it is a technical issue of only marginal importance. However, the discussed practices of testing latent structures (especially the disregard for the model test in combination with an uncritical acceptance of the approximate fit hypothesis without any examination of relevant evidence) can have far-reaching consequences, since they weaken the ability of the data to contradict the postulated models (see Barrett, 2007; McIntosh, 2007; Millsap, 2007; Kline, 2011; Hayduk, 2014b, for discussion). Moreover, the current reproducibility crisis (Open Science and Collaboration, 2015) has shown that such issues cannot be regarded peripheral since they lie right at the heart of credibility of science as such.

As one may have guessed, it is unrealistic to expect that the verification of a theory postulating complex interrelationships between latent and manifested variables is a matter of a single test and a bunch of indices. In fact, a significant χ^2^ does not necessarily imply a useless model – just as satisfactory AFI do not imply that the model is roughly true. It is always the (partly subjective) judgment informed by data that should act as a referee (Mulaik, 2009). In concord with the Popperian logic, it can be concluded that the confirmatory latent techniques cannot truly verify models, but they have the capability of digging up empirical evidence of problems in these models (see Hayduk, 2014b). If this accept-support statistical paradigm gets robbed of its strictness by the loosely applied dogma of approximate fit, it loses its scientific value of pushing the researcher to look for eventual flaws of the postulated theory in order to improve it. The findings of this study indicate that researchers generally tend to regard their models as an adequate representation of the data, irrespective of model test outcome, since as many as 80% of these accepted models failed in the face of statistical testing. At the same time, in just 3% of the studies did the authors set out to examine the evidence concerning the triviality of model misspecification (i.e., the inspection of the residual matrix) and none verified the claim about excessive power of the model test. In the light of these arguments, it leads to a perilous question: Does it make sense to statistically confront the theoretical models with the data provided that no consequences exist, i.e., that the model is accepted even after failing the statistical test of fit to the data? Unfortunately, the pragmatic answer might be no, testing with no consequences is useless. And that is the danger here – discarding model testing – the primary means of quantitative inference without any real alternative.

Hopefully, this non-technical reflection will lead to more critical insight into the issues of latent modeling techniques and prompt researchers to more rigorously test and carefully inspect (and not just confirm) modeled latent structures.

## Conflict of Interest Statement

The author declares that the research was conducted in the absence of any commercial or financial relationships that could be construed as a potential conflict of interest.

## References

[B1] Antonakis J. Bendahan S. Jacquart P. Lalive R. (2010). On making causal claims: a review and recommendations. *Leader. Q.* 21 1086–1120. 10.1016/j.leaqua.2010.10.010

[B2] Asparouhov T. Muthén B. (2009). Exploratory structural equation modeling. *Struct. Equ. Model.* 16 397–438. 10.1080/10705510903008204

[B3] Barrett P. (2007). Structural equation modeling: adjudging model fit. *Pers. Individ. Dif.* 42 815–824. 10.1016/j.paid.2006.09.018

[B4] Beauducel A. Wittmann W. (2005). Simulation study on fit indices in CFA based on data with slightly distorted simple structure. *Struct. Equ. Model.* 12 41–75. 10.1207/s15328007sem1201_3

[B5] Bollen K. A. (1989). *Structural Equations with Latent Variables.* New York: Wiley.

[B6] Bollen K. A. Kirby J. B. Curran P. J. Paxton P. M. Chen F. N. (2007). Latent variable models under misspecification — Two-stage least squares (2SLS) and maximum likelihood (ML) estimators. *Sociolog. Methods Res.* 36 48–86. 10.1177/0049124107301947

[B7] Boomsma A. Hoyle R. H. Panter A. T. (2012). “The structural equation modeling research report,” in *Handbook of Structural Equation Modeling*, ed. Hoyle R. H. (New York, NY: Guilford Press), 341–358.

[B8] Borsboom D. (2005). *Measuring the Mind: Conceptual Issues in Contemporary Psychometrics.* Cambridge: Cambridge University Press.

[B9] Borsboom D. Mellenbergh G. J. Van Heerden J. (2003). The theoretical status of latent variables. *Psychol. Rev.* 110 203–219. 10.1037/0033-295X.110.2.203 12747522

[B10] Breckler S. J. (1990). Applications of covariance structure modeling in psychology – cause for concern. *Psychol. Bull.* 107 260–273. 10.1037//0033-2909.107.2.260 2320704

[B11] Browne M. W. Cudeck R. (1992). Alternative ways of assessing models? *Sociol. Methods Res.* 21 230–258. 10.1177/0049124192021002005

[B12] Chen F. Bollen K. A. Paxton P. Curran P. Kirby J. (2001). Improper solutions in structural equation models: causes, consequences, and strategies. *Sociol. Methods Res.* 29 468–508. 10.1177/0049124101029004003

[B13] DiStefano C. Hess B. (2005). Using confirmatory factor analysis for construct validation: an empirical review. *J. Psychoeduc. Assess.* 23 225–241. 10.1177/073428290502300303

[B14] Fan X. Sivo S. A. (2005). Sensitivity of fit indices to misspecified structural or measurement model components: rationale of two–index strategy revisited. *Struct. Equ. Model.* 12 343–367. 10.1207/s15328007sem1203_1

[B15] Fan X. Thompson B. Wang L. (1999). The effects of sample size, estimation methods, and model specification on SEM fit indices. *Struct. Equ. Model.* 6 56–83. 10.1080/10705519909540119

[B16] Fouladi R. T. (2000). Performance of modified test statistics in covariance and correlation structure analysis under conditions of multivariate nonnormality. *Struct. Equ. Model.* 7 356–410. 10.1207/S15328007SEM0703_2

[B17] Guo B. Perron B. E. Gillespie D. F. (2009). A systematic review of structural equation modelling in social work research. *Br. J. Soc. Work* 39 1556–1574. 10.1093/bjsw/bcn101 25477696 PMC4251473

[B18] Hayduk L. A. (2014a). Seeing perfectly-fitting factor models that are causally misspecified: understanding that close-fitting models can be worse. *Educ. Psychol. Measure.* 74 905–926. 10.1177/0013164414527449

[B19] Hayduk L. A. (2014b). Shame for disrespecting evidence: the personal consequences of insufficient respect for structural equation model testing. *BMC Med. Res. Method.* 14:124 10.1186/1471-2288-14-124 PMC4297459 25430437

[B20] Hayduk L. A. Cummings G. Boadu K. Pazderka-Robinson H. Boulianne S. (2007). Testing! Testing! one, two, three – testing the theory in structural equation models! *Pers. Individ. Dif.* 42 841–850. 10.1016/j.paid.2006.10.001

[B21] Hayduk L. A. Glaser D. N. (2000). Jiving the four–step, waltzing around factor analysis, and other serious fun. *Struct. Equ. Model.* 7 1–35. 10.1207/S15328007SEM0701_01

[B22] Hayduk L. A. Pazderka-Robinson H. Cummings G. Levers M. J. Beres M. A. (2005). Structural equation model testing and quality of natural killer cell activity measurements. *BMC Med. Res. Method.* 5:1 10.1186/1471-2288-5-1 PMC546216 15636638

[B23] Heene M. Hilbert S. Draxler C. Ziegler M. Bühner M. (2011). Masking misfit in confirmatory factor analysis by increasing unique variances: a cautionary note on the usefulness of cutoff values of fit indices. *Psychol. Methods* 16 319–336. 10.1037/a0024917 21843002

[B24] Heene M. Hilbert S. Freudenthaler H. H. Bühner M. (2012). Sensitivity of SEM fit indexes with respect to violations of uncorrelated errors. *Struct. Equ. Model.* 19 36–50. 10.1080/10705511.2012.634710

[B25] Hershberger S. L. (2003). The growth of structural equation modeling: 1994–2001. *Struct. Equ. Model.* 10 35–46. 10.1207/S15328007SEM1001_2

[B26] Hooper D. Coughlan J. Mullen M. R. (2008). Structural equation modeling: guidelines for determining model fit. *Elec. J. Business Res. Methods* 6 53–60.

[B27] Hu L. Bentler P. M. (1999). Cutoff criteria for fit indexes in covariance structure analysis: conventional criteria versus new alternatives. *Struct. Equ. Model.* 6 1–55. 10.1080/10705519909540118

[B28] Jackson D. L. Gillaspy J. A. Purc-Stephenson R. (2009). Reporting practices in confirmatory factor analysis: an overview and some recommendations. *Psychol. Methods* 14 6–23. 10.1037/a0014694 19271845

[B29] Kaplan D. (1988). The impact of specification error on the estimation, testing, and improvement of structural equation models. *Multivariate Behav. Res.* 23 69–86. 10.1207/s15327906mbr2301_4 26782258

[B30] Kirby J. B. Bollen K. A. (2009). Using instrumental variable (IV) tests to evaluate model specification in latent variable structural equation models. *Sociol. Methodol.* 39 327–355. 10.1111/j.1467-9531.2009.01217.x 20419054 PMC2858448

[B31] Kline R. B. (2011). *Principles and Practice of Structural Equation Modeling.* New York: Guilford Press.

[B32] MacCallum R. C. Browne M. W. Sugawara H. M. (1996). Power analysis and determination of sample size for covariance structure modeling. *Psychol. Methods* 1 130–149. 10.1037/1082-989X.1.2.130

[B33] MacCallum R. C. Roznowski M. Necowitz L. B. (1992). Model modifications in covariance structure analysis: the problem of capitalization on chance. *Psychol. Bull.* 111 490–504. 10.1037/0033-2909.111.3.490 16250105

[B34] Marsh H. W. Hau K. T. Wen Z. (2004). In search of golden rules: comment on hypothesis–testing approaches to setting cutoff values for fit indexes and dangers in overgeneralizing Hu and Bentler’s (1999) findings. *Struct. Equ. Model.* 11 320–341. 10.1207/s15328007sem1103_2

[B35] Marsh H. W. Morin A. J. S. Parker P. D. Kaur G. (2014). Exploratory structural equation modeling: an integration of the best features of exploratory and confirmatory factor analysis. *Annu. Rev. Clin. Psychol.* 10 85–110. 10.1146/annurev-clinpsy-032813-153700 24313568

[B36] McIntosh C. N. (2007). Rethinking fit assessment in structural equation modeling: a commentary and elaboration on Barrett. *Pers. Individ. Dif.* 42 859–867. 10.1016/j.paid.2006.09.020

[B37] McIntosh C. N. (2012). Improving the evaluation of model fit in confirmatory factor analysis. *Qual. Life Res.* 21 1619–1621. 10.1007/s11136-011-0084-4 22249309

[B38] Miles J. Shevlin M. (2007). A time and a place for incremental fit indices. *Pers. Individ. Dif.* 42 869–874. 10.1016/j.paid.2006.09.022

[B39] Millsap R. E. (2007). Structural equation modeling made difficult. *Pers. Individ. Dif.* 42 875–881. 10.1016/j.paid.2006.09.021

[B40] Moshagen M. (2012). The model size effect in SEM: inflated goodness–of–fit statistics are due to the size of the covariance matrix. *Struct. Equ. Model.* 19 86–98. 10.1080/10705511.2012.634724

[B41] Mulaik S. A. (2001). The curve–fitting problem: an objectivist view. *Philos. Sci.* 68 218–241. 10.1086/392874

[B42] Mulaik S. A. (2009). *Linear Causal Modeling with Structural Equations.* New York: CRC Press.

[B43] Mulaik S. A. James L. R. Van Alstine J. Bennett N. Lind S. Stillwell C. D. (1989). An evaluation of goodness–of–fit indices for structural equation models. *Psychol. Bull.* 105 430–445. 10.1037/0033-2909.105.3.430

[B44] Muthén L. K. Muthén B. O. (2002). How to use a Monte Carlo study to decide on sample size and determine power. *Struct. Equ. Model.* 4 599–620. 10.1207/S15328007SEM0904_8

[B45] Nunkoo R. Ramkissoon H. Gursoy D. (2013). Use of structural equation modeling in tourism research: past, present, and future. *J. Travel Res.* 52 759–771. 10.1177/0047287513478503

[B46] Olsson U. H. Foss T. Troye S. V. Howell R. D. (2000). The performance of ML. GLS, and WLS estimation in structural equation modeling under conditions of misspecification and non–normality. *Struct. Equ. Model.* 7 557–595. 10.1207/S15328007SEM0704_3

[B47] Open Science and Collaboration (2015). Estimating the reproducibility of psychological science. *Science* 349:aac4716 10.1126/science.aac4716 26315443

[B48] Raykov T. Penev S. (1999). On structural equation model equivalence. *Multivariate Behav. Res.* 34 199–244. 10.1207/S15327906Mb340204 26753936

[B49] Saris W. E. den Ronden J. Satorra A. (1985). “Testing structural equation models,” in *Structural Modeling by Example*, eds Cuttance P. F. Ecob J. R. (Cambridge: Cambridge University Press), 202–220.

[B50] Saris W. E. Satorra A. (1993). “Power evaluations in structural equation models,” in *Testing Structural Equation Models*, eds Bollen K. A. Long J. S. (Newbury Park: Sage), 181–204.

[B51] Saris W. E. Satorra A. van der Veld W. (2009). Testing structural equation models or detection of misspecifications? *Struct. Equ. Model.* 16 561–582. 10.1080/10705510903203433

[B52] SCImago (2007). *SJR — SCImago Journal and Country Rank.* Available at: http://www.scimagojr.com

[B53] Sharma S. Mukherjee S. Kumar A. Dillon W. R. (2005). A simulation study to investigate the use of cutoff values for assessing model fit in covariance structure models. *J. Business Res.* 58 935–943. 10.1016/j.jbusres.2003.10.007

[B54] Shipley B. (2003). Testing recursive path models with correlated errors using d-separation. *Struct. Equ. Model.* 10 214–221. 10.1207/S15328007SEM1002_3

[B55] Steiger J. H. (2000). Point estimation, hypothesis testing, and interval estimation using the RMSEA: some comments and a reply to Hayduk and Glaser. *Struct. Equ. Model.* 7 149–162. 10.1207/S15328007SEM0702_1

[B56] Tomarken A. J. Waller N. G. (2003). Potential problems with “well–fitting” models. *J. Abnorm. Psychol.* 112 578–598. 10.1037/0021-843X.112.4.578 14674870

[B57] Vernon T. Eysenck S. B. G.(eds). (2007). Special issue on structural equation modeling [Special issue]. *Pers. Individ. Dif.* 42 811–898.

[B58] Wheaton B. (1987). Assessment of fit in overidentified models with latent variables. *Sociol. Methods Res.* 16 118–154. 10.1177/0049124187016001005

[B59] Yuan K. H. (2005). Fit indices versus test statistics. *Multivariate Behav. Res.* 40 115–148. 10.1207/s15327906mbr4001_5 26822275

[B60] Yuan K. H. Bentler P. M. Zhang W. (2005). The effect of skewness and kurtosis on mean and covariance structure analysis: the univariate case and its multivariate implication. *Sociol. Methods Res.* 34 240–258. 10.1177/0049124105280200

