# Corrigendum: A cautionary note on testing latent variable models

**DOI:** 10.3389/fpsyg.2017.00168

**Published:** 2017-02-07

**Authors:** Ivan Ropovik

**Affiliations:** Department of Preschool and Elementary Education and Psychology, Faculty of Education, University of PresovPresov, Slovakia

**Keywords:** structural equation modeling, confirmatory factor analysis, model fit, chi square test, approximate fit indices

In the original article, there was an error. In two places, “>” and “ < ” operators related to *p*-values had been inadvertently substituted, stating *p* > 0.05 when in fact *p* < 0.05.

A correction has been made to the Introduction, second paragraph:

To put it simply, this (dis)confirmatory technique allows testing of several postulated hypotheses simultaneously. Employing iterative estimation, it tries to find a unique set of implied parameters (variances, covariances) that would match those in the matrix of observed covariances as much as possible. In such a manner, it can be tested whether the model-implied relations between latent and manifested variables correspond with the existing relations observed in the data. Within SEM, the only statistical test of model-data fit is the *chi-square test* (χ^2^, actually, a family of tests). It tests the null hypothesis that the model-implied covariance matrix Σ(**θ**) does not significantly differ from the matrix of observed covariances **S**, i.e., that the residuals are not statistically different than zero. A high value of χ^2^ (relative to the model's *df*) associated with *p* < 0.05 means the following: Given that the null hypothesis of no model-data difference is true, the observed discrepancies between the model and the data are too big to be caused by random fluctuations due to sampling error alone, indicating the presence of a systematic misspecification in the tested model. Logically, following such an indication, the researcher should try to find the misspecification errors that are present in the model in order to achieve convergence between scientific explanation and the principle of phenomena under study.

This has also been corrected in Results section, third paragraph:

Among the most important aspects of every analysis are the consequences of model testing and the interpretation of the model (does the model fit the data so that it is possible to interpret model parameters?). Here, almost all of the studies (97%) reported at least one model that was considered adequate and served as the basis for further interpretations. However, out of these models (*N* = 73), 80% did not fit according to the model test and the decision to retain and interpret the model was probably based on some other criteria, particularly AFI (40% of these studies just provided the fit indices and noted that the model fits but did not explicate the basis for such conclusion). Only 3% of the studies (*N* = 2) concluded that the best model does not fit by any measures, however, one of them proceeded to the interpretation of model parameters anyway. Overall, 80% of the studies ignored the χ^2^ model test either by ignoring the associated significance of *p* < 0.05, or by not reporting it at all. Out of these studies (*N* = 60), 75% did not mention the reasons for ignoring the model test. On the other hand, the explicitly stated reasons given by the authors for ignoring the outcome of the model test can be summarized as follows: (1) χ^2^ is overly sensitive to sample size, (2) χ^2^ penalizes models when the number of variables gets high, (3) the exact fit hypothesis is nonsensical and (4) there is a broad consensus on the preference of the use of AFI.

The author apologizes for this error and state that this does not change the scientific conclusions of the article in any way.

## Conflict of interest statement

The author declares that the research was conducted in the absence of any commercial or financial relationships that could be construed as a potential conflict of interest.

